# The bright and the dark side of team diversity: Uncovering the effective functioning drivers

**DOI:** 10.1108/JHOM-09-2025-0626

**Published:** 2026-07-10

**Authors:** Davide Trinchese, Francesca Pennucci, Milena Vainieri

**Affiliations:** Management and Healthcare Laboratory (MeS), Sant'Anna School of Advanced Studies, Institute of Management, Pisa, Italy

**Keywords:** Team diversity, Team functioning, Healthcare teams, Social cognitive theory, Employee relations

## Abstract

**Purpose:**

This study aims to investigate when and how diversity has a positive impact on the effective functioning of the teams and when and how it leads to team dysfunctionality. It explores the strategic drivers that influence team dynamics on the basis of the Social Cognitive Theory.

**Design/methodology/approach:**

Qualitative in-depth interviews with Italian healthcare employees were conducted to collect empirical experiences from 15 key informants working in and with healthcare diversified teams. The data were interpreted using reflexive thematic analysis, which facilitated the exploration and identification of core strategic drivers connected to team diversity.

**Findings:**

This study, in light of the social cognitive theory, underscores the value of diversity in mixing competencies, perspectives, and resources to address complex challenges, particularly in healthcare settings. However, it also highlights that diversity can become counterproductive when team members lack shared goals or alignment, resulting in miscommunication, poor coordination, and overall dysfunction. In this scenario, six themes emerged from the thematic analysis: (1) task-related; (2) biodemographic functionalities and dysfunctionalities; (3) leaders vs free riders; (4) external environment dynamics; (5) the supervisor's role; (6) the temporal dynamics of team diversity.

**Originality/value:**

This study contributes to the existing literature on team dynamics as one of the few qualitative investigations specifically aimed at understanding when team diversity becomes functional or dysfunctional. Theoretically, it provides qualitative evidence grounded in the social cognitive theory, whereas practically, the study offers actionable strategies for managers, highlighting the key strategic drivers that can either prevent or foster team dysfunction.

**Highlights:**

1. Team diversity can lead to either positive or negative outcomes, depending on specific team dynamics.
2. Diversity alone is not enough; shared goals, mutual trust, and clear communication are essential to prevent dysfunction and counterproductive behaviors such as free riding.
3. Several drivers influence team functionality, including the supervisor's role, external macro-level dynamics, and the impact of time.
4. Social Cognitive Theory offers a dynamic framework to understand how diversity impacts team functionality or dysfunctionality, emphasizing the reciprocal interaction of personal factors, behavioral patterns, and environmental influences on individuals in shaping positive or negative outcomes through mechanisms like observational learning, self-efficacy, and outcome expectations.

## Introduction

The composition of teams is increasingly driven towards diversity. Due to the high complexity of current processes and issues, policymakers and human resources managers are pushing for the formation of diversified teams capable of addressing a wide range of multi-sided needs through the intersection of different competencies (Homberg and Bui, 2013). In this respect, more research on team composition is becoming prominent, especially from an empirical point of view (Bunderson and Sutcliffe, 2002; Buyl *et al*., 2011; Homberg and Bui, 2013; Qian *et al*., 2013). Mixed evidence exists regarding the positive impact of diversity on teamwork (Bunderson and Sutcliffe, 2002; Buyl *et al*., 2011; Mitchell *et al*., 2020). An important assumption underlying much of the debate is that heterogeneity in teams, if not properly managed, leads to dysfunctionality, including conflict, mistrust, and inefficiency. While this assumption is reasonable and supported by meta-analyses and empirical studies (Horwitz and Horwitz, 2007; Tajfel *et al*., 1979; van Knippenberg *et al*., 2004; Webber and Donahue, 2001), it is rarely problematized or critically questioned in depth. In this sense, this paper aims to provide evidence on how diversity can be managed effectively by mitigating dysfunctional behaviors in diverse teams. Indeed, it is fundamental to better define the levers and barriers to building functional teams, rather than just *groups of people* (Peduzzi, 2001). In this paper, we explicitly position ourselves within this ongoing debate, aiming to refine understanding of how and when diversity becomes functional or dysfunctional in teamwork. To do so, we adopt a broad definition of diversity and, following Horwitz and Horwitz (2007), categorize it into biodemographic and task-related diversity.

Biodemographic diversity represents innate member characteristics that are immediately observable and categorized (e.g. age, gender, and race/ethnicity) whereas task-related diversity is acquired individual attributes (e.g. functional expertise, education, and organizational tenure) that have been postulated to be more germane to accomplishing tasks than biodemographic diversity (Horwitz and Horwitz, 2007, p. 990).

Consequently, we examined the potential functional and dysfunctional effects of diversity, such as communication barriers, conflict, and inefficiencies, offering a balanced view of its impact (Leslie, 2019; Von Bergen *et al*., 2002). To provide a comprehensive theoretical foundation for understanding these dynamics, this study draws upon Social Cognitive Theory (SCT), which offers valuable insights through its core principle of reciprocal determinism (Bandura, 1999, 2001, 2011, 2014). According to SCT, outcomes emerge from the continuous interaction between personal factors (individual competencies, characteristics, and beliefs), behavioral patterns (collaboration styles, communication approaches, and conflict resolution strategies), and environmental influences (organizational context, external pressures, and structural constraints). This theoretical lens helps to explain why diversity sometimes leads to functionality and other times results in dysfunction, as these three determinants continuously shape and reshape team dynamics. Moreover, we identified several drivers that can affect diversity and its effective functioning in teams. We conceptualize diversity not simply as a static composition of team members but as a relational and dynamic construct embedded in the interplay of individual attributes, interpersonal interactions, and organizational context. This conceptualization aligns with the SCT understanding of human behavior as emerging from reciprocal interactions rather than from isolated individual or environmental factors (Andersen and Chen, 2002). The study focuses on the healthcare sector, characterized by multilevel organizations, high interdependence among professionals, the need for rapid decision-making, and multidisciplinary collaboration to address patients' diverse needs. In this context, the primary motivation for selection lies in its intrinsic complexity and the crucial importance of team in ensuring effective health outcomes and sustainable use of resources (Borrill *et al*., 2000; Leggat, 2007; Rosen *et al*., 2018). This makes it a fertile ground for studying the dynamics of diversity within teams and its implications (Johnson *et al*., 2018). In this sense, this evidence-based study aligns with broader theoretical developments that conceptualize diversity as multidimensional and context-dependent (Harrison and Klein, 2007) and responds to recent efforts to better capture complexity and organizational dynamics (Nkomo *et al*., 2019). By engaging with these perspectives and applying them to healthcare delivery organizations, this study aims to bridge the gap between theoretical discussions on diversity and the practical challenges of managing diverse workforces in high-stakes service environments. More specifically, it contributes to the literature by empirically examining contextual factors that are widely acknowledged as crucial for understanding diversity processes but have rarely been systematically incorporated into a practical context. Building on social cognitive theory, the study integrates these contextual contingencies, thereby offering an innovative, evidence-based account of how diversity unfolds in organizational settings.

## Background

### The bright and the dark side of team diversity: a short review

Mixed evidence is available on the role of team diversity, and different theories have been used to interpret its effects. According to resource dependence theory, the greater the diversity within a group, the more the people with complementary human and social capital will be present, fostering interconnections spanning the organization, its employees, and the environment (Drees and Heugens, 2013; Hillman *et al*., 2009; Salancik and Pfeffer, 1978). The information-decision-making perspective highlights the positive impact of diversity on decision-making (Bantel and Jackson, 1989; Seong and Hong, 2018; van Knippenberg *et al*., 2004). According to this viewpoint, the quality of decisions is influenced by the exchange of information within a team and how that information is processed. Diverse groups can draw on a wide array of task-related knowledge, skills, and abilities, benefiting from the unique perspectives and ideas that members bring (Gebert *et al*., 2010; Hinsz *et al*., 1997). In this sense, “team diversity leads to broader perspectives and a greater amount of information shared, consequently enhancing decision quality” (Homberg and Bui, 2013, p. 458).

Conversely, social identity theory views diversity as a factor that divides workers into subgroups, hindering decision-making and ultimately reducing organizational outcomes (Tajfel *et al*., 1979). In this respect, George (1990) suggested that team emotions evolve as organizations become more alike over time. This occurs because individuals are attracted to teams whose members share similar emotional tendencies. Existing team members also tend to choose new members who reflect their own emotional orientations. Consequently, over time, team members who align with the team's emotional norms are more likely to remain, while those who differ are more likely to depart. This process leads teams to become more alike in their emotional characteristics over time. In this sense, according to Allport *et al*. (1954), when individuals form new groups, they tend to minimize uncertainty that arises from not knowing their team members well, in order to avoid potential conflicts in relationships. The diversity among team members often triggers feelings of fear and uncertainty. Hence, following this view, cohesion and decision quality of teams are positively influenced by the similarity among their members, which guarantees a strategic advantage (Michel and Hambrick, 1992; van Knippenberg *et al*., 2004).

Despite considerable efforts in the literature to understand the actual impact of team diversity on various organizational outcomes, the evidence remains mixed. (Buyl *et al*., 2011; Homberg and Bui, 2013; Jansen and Searle, 2021). One of the main examples of this complexity in reaching an effective answer is identified by Horwitz and Horwitz (2007) which conducted a meta-analysis of the main studies on the topic and found a positive relationship between task-related diversity and performance, and a non-significant relationship between biodemographic diversity and performance, portraying diversity *“as a ‘double-edged sword’ in contemporary organizational theory”* (Horwitz and Horwitz, 2007, p. 998), see also (Jansen and Searle, 2021).

In this scenario, we focus our attention on the healthcare sector. The healthcare sector has undergone significant and transformative changes in recent years, largely driven by socio-demographic shifts that have reshaped healthcare delivery. One of the primary factors behind these changes has been the increase in life expectancy, which, coupled with the rise in chronic diseases, has placed new and substantial demands on healthcare systems (Beltrán-Sánchez *et al*., 2015). These shifts have not only impacted patient care but also led to significant changes in the structure of healthcare teams and the roles of healthcare professionals. At the same time, the healthcare sector has faced a persistent shortage of key professionals, including physicians, nurses, and other healthcare workers, which has compounded these challenges (Heilmann, 2010; Okyere *et al*., 2017). As a result, healthcare organizations have had to adopt new strategies to maintain care quality and efficiency, despite the growing gap between demand and workforce capacity. This imbalance has triggered a shift in how care is provided, with a stronger emphasis on diversification within healthcare teams to address patients' varying needs (Gomez and Bernet, 2019). However, while diversity has driven greater specialization and efficiency, it also needs to be balanced by the need to form flexible, adaptable teams that can effectively meet patients' diverse needs (Grace *et al*., 2014). Hence, diversity within healthcare teams has been perceived as functional in some cases and dysfunctional in others (Hofhuis *et al*., 2018; Johnson *et al*., 2018). The main reason for this lies in the fact that “diverse healthcare teams are becoming more common as healthcare organizations move towards a holistic model of patient care, we need to have a better understanding of when and which team processes matter most for healthcare teams to be effective (Johnson *et al*., 2018, p. 443).” In this respect, healthcare is one of the most interesting sectors for understanding the dynamics that lead to team functionality or dysfunctionality.

### Conceptual framework

To better understand this “double-edged sword” phenomenon and provide a coherent theoretical foundation for our research, we adopt SCT as our primary conceptual framework. According to SCT, “behavior, cognitive, and other personal factors and environmental events operate as interacting determinants that influence each other bidirectionally” (Wood and Bandura, 1989, p. 362). This triadic model is particularly relevant for understanding the effective functioning of team diversity because it recognizes that individual responses are not predetermined but rather emerge through ongoing interactions between personal cognitive processes, observable behaviors, and contextual factors. Applied to team diversity, there can be conceptualized critical mechanisms that determine whether outcomes are functional or dysfunctional.

Observational learning allows to understand how diversity effects emerge in practice. Team members continuously observe how leaders and colleagues interact across differences, learning appropriate collaborative behaviors through these interactions rather than through formal training alone (Bandura and Walters, 1977; Fryling *et al*., 2011). This learning process is particularly powerful because it provides concrete behavioral scripts for navigating diversity challenges. When team members witness positive models of inclusion, constructive conflict resolution, and cross-functional cooperation, they can internalize these patterns and replicate them in their own interactions (Edmondson, 1999). In this sense, “increasing diversity is likely to increase the cognitive resources, through bringing together members with different perspectives, approaches, knowledge, skills and experiences, and also emotional/social demands, as diverse teams require members to deal with people who are different to themselves (Jansen and Searle, 2021, p. 1848)”. Contrarily, when employees observe exclusionary behaviors, stereotyping, or avoidance of diverse perspectives, these negative patterns become normalized and perpetuated throughout the team system.

Self-efficacy refers to individual beliefs about performing a task successfully in a particular situation, while group efficacy encompasses shared beliefs about the team's ability to leverage differences constructively (Bandura, 1997; Gibson, 2001). In our framework, these efficacy beliefs fundamentally determine whether team members perceive heterogeneity as a valuable resource that enhances problem-solving capacity or as a threatening challenge that complicates coordination and communication. Research underlines that teams with higher collective efficacy are significantly better able to integrate diverse professional perspectives and achieve superior outcomes (Shin *et al*., 2012; Watson *et al*., 2001).

Outcome expectations create self-fulfilling prophecies that either facilitate or undermine collaborative potential. When team members hold positive diversity beliefs, they actively seek out different perspectives, engage in constructive debate, and invest effort in integration processes (Homan *et al*., 2008; Van Knippenberg *et al*., 2013). Conversely, negative expectations lead to protective behaviors, communication avoidance, and attribution biases that systematically undermine team functioning (Bezrukova *et al*., 2016; Chatman and Flynn, 2001).

Environmental context, including organizational structures, leadership styles, communication processes, and cultural norms, creates the broader social environment that either facilitates or constrains positive diversity expression (Kozlowski and Ilgen, 2006). SCT emphasizes that individual cognitions and behaviors are embedded within and shaped by these contextual factors through ongoing reciprocal influence. This theoretical lens explains why the same diversity characteristics can produce “bright side” effects in some contexts while generating “dark side” outcomes in others. The difference lies not in the diversity itself but in the social-cognitive processes that emerge from the interaction among individual, behavioral, and environmental factors (Guillaume *et al*., 2017; Mitchell *et al*., 2015). SCT thus provides a dynamic, process-oriented framework for understanding diversity outcomes that goes beyond simple input-output models to examine the mediating cognitive and social mechanisms.

Based on these premises, this research aims to understand how these contextual processes influence team diversity, both functionally and dysfunctionally, in healthcare settings.

RQ.
How does diversity within healthcare teams impact their functionality and dysfunctionality, and what are the key social cognitive dynamics that determine these effects?

SCT directly justifies our qualitative research approach based on in-depth interviews with healthcare managers. Since diversity outcomes emerge through cognitive interpretations, social learning processes, and subjective experiences within specific contexts, understanding these dynamics requires exploring the lived experiences of individuals who manage diverse teams. Through interviews, we can examine how employees perceive the effects of diversity, the behavioral models they observe, how they develop and maintain efficacy beliefs, and the strategies they employ to enhance collective efficacy and positive outcome expectations.

## Material and methods

The paper presents a qualitative study, conducted with in-depth online semi-structured interviews. A purposive sample of top and middle managers was selected to capture evidence on their experience of team diversity and team functionality in the healthcare sector.

The participants were selected according to the following criteria: (1) worked in the healthcare sector, (2) held managerial roles, and (3) were members of teams before being managers. The sampling aimed at including participants who could provide comparative insights into varying social cognitive dynamics that they experienced about diversity management across different managerial contexts and time periods. We aimed for diversity in gender, age, and experience, with respect to level of responsibility and job setting. 15 respondents were recruited from November 2024 to June 2025, 7 middle managers and 8 top managers with an average age of 58,7 (range 49–70) and 30 years of experience (range 17–50). The majority of interviewees were male (10 out of 15), as expected given the current composition of the middle and top management group in Italy (Trinchese *et al*., 2024).

The interview protocol was deliberately constructed to operationalize the core mechanisms of SCT, ensuring direct alignment between our theoretical framework and data collection strategy (Maxwell, 2012). Following SCT's emphasis on “reciprocal interplay of intrapersonal, behavioral, and environmental determinants (Bandura, 2006, p. 165)”, the first group of questions were designed to explore: (1) personal; (2) behavioral patterns and (3) environmental influences on the basis of the lived experience of the employees. In this sense, the questions examined were: (1) Definition of team and teamwork; (2) Experience in diversified teams; (3) Factors related to diversity; (4) Functionalities associated with diversity in teams; (5) Dysfunctionalities associated with diversity in teams. The second set of questions had the goal of determining which are the key dynamics related to team diversity and which are the levers for managers: (1) Key dynamics associated with diversity; (2) Individual-level levers; (3) Group-level levers; (4) Suggestions for future managers.

Table 1 presents the domain, rationale, and references for including these topics in the interviews. It is designed to provide clarity on why these areas were emphasized in the interviews and the academic foundations that support their relevance.

**Table 1:** In-depth semi-structured interview: domain, rationale, and references

Domain	Rationale	References
Definition of team and teamwork	A clear definition of what constitutes a team and the purpose of teamwork is essential for introducing interviewees to the argument. Misalignment in this definition leads to inconsistent expectations and poor team functioning	Bantel and Jackson (1989), Borrill *et al*. (2000), Leggat (2007), Rosen *et al*. (2018), Gebert *et al*. (2010), Peduzzi (2001) Grace *et al*. (2014)
Experience in diversified teams	Different experiences within the team can, on one hand, lead to viewing a diversified team as a valuable resource or a signal of the organization's reputation. On the other hand, it could lead to perceiving diversity as an element that undermines communication, trust, and alignment, thereby having a negative impact on the organization's outcomes. Moreover, the living experience of people members allows to analyze team diversity in the light of the triadic interaction among personal factors, behavioral patterns, and environmental influence elucidated by SCT	Salancik and Pfeffer (1978), van Knippenberg *et al*. (2004), Tajfel *et al*. (1979), Horwitz and Horwitz (2007), Bandura (2014, 2011, 2001, 1999)
Factors related to diversity	The study defines diversity as both observable or readily detectable attributes (such as age, gender, nationality) and less visible or underlying attributes (such as personality, cognitive styles, cultural background, educational background, functional background), which help to go deeper into the relationship between SCT triadic interactions and team diversity	Martins and Sohn (2022), Horwitz and Horwitz (2007), Jansen and Searle (2021)
Functionalities associated with diversity in teams	A diverse team can enhance creativity, innovation, and adaptability, helping the team address complex problems. The key is to identify the positive side of the drivers that allow to assign the right functions to the different employees, valuing the diverse attributes of the team members, and exploiting in an effective way the SCT triadic interactions	Bunderson and Sutcliffe (2002), Homberg and Bui (2013)
Dysfunctionalities associated with diversity in teams	A diverse team can lead to a lack of cohesion, conflicting values, and poor communication. The key is to identify the negative side of the drivers, which does not allow for the exploitation in a synergistic way of the SCT triadic interactions. Diversity may introduce challenges in such scenarios, such as conflict, inefficiency, or exclusion, ultimately resulting in negative outcomes	Leslie (2019), Von Bergen *et al*. (2002)
Key dynamics related to diversity and suggestions for the management of diversity	The rationale behind this area is to understand the key dynamics that foster or hinder the functionality of team diversity, and to identify possible actions that policymakers and human resources managers can take to make a diverse team functional (exploiting in a synergistic way the SCT triadic interactions and consequently the drivers identified) rather than dysfunctional	

**Source(s):** Authors’ own work

### Data collection

Video and audio recordings were collected with participants' consent and later transcribed verbatim. Two trained interviewers participated in the online sessions, with one leading the interview and the other taking notes. Each interview lasted approximately 40 min, and all participants agreed to be recorded. In compliance with GDPR, individuals granted verbal informed consent to the use of their statements and video recordings. Participants were also informed that they could access, edit, or delete their transcripts at any time.

Data collection was conducted using purposive sampling rather than solely relying on saturation (Gentles *et al*., 2015). In fact, the decision to conclude the data collection process was also guided by the concept of data saturation, which can be defined as the point at which the research team stops observing new information or themes in the data, indicating that additional data collection is unlikely to yield different or new results (Etikan, 2016; Guest *et al*., 2006; Tongco, 2007). Importantly, our findings align with the position of Saunders *et al*. (2018) that emphasize how saturation is not a strict numerical threshold but rather a matter of thematic completeness. They argue that saturation can be achieved with a relatively small number of interviews when the research context is clearly defined and the participant group is purposively selected.

The complete list of interviews and respondents' characteristics is reported in Table 2.

**Table 2:** In-depth semi-structured interview: current managerial role, gender, age, experience and professional background

*n*	Current managerial role	Gender	Age	Years of experience in healthcare	Professional background
I.1	Top Manager	M	69	42	Pediatrician
I.2	Top Manager	M	61	27	Public Health Physician
I.3	Middle Manager	F	62	39	Social Worker
I.4	Middle Manager	F	63	19	Communication Studies
I.5	Top Manager	M	60	40	Jurist
I.6	Top Manager	M	50	20	Public Health Physician
I.7	Top Manager	M	70	50	Oncological Gynecologist
I.8	Top Manager	M	50	20	Economist
I.9	Top Manager	M	55	26	Economist
I.10	Middle Manager	M	52	30	Nurse
I.11	Middle Manager	F	58	38	Nurse
I.12	Middle Manager	M	61	21	Humanist
I.13	Middle Manager	F	49	17	Communication Studies
I.14	Top Manager	F	64	39	Social Worker
I.15	Middle Manager	M	61	25	Historian

**Source(s):** Authors’ own work

### Data analysis

The original transcriptions of each interview were analyzed by two researchers independently after anonymization, in which each participant was assigned a code. To address potential concerns about self-fulfilling prophecy in our analytical approach, we implemented a rigorous multi-stage coding strategy that moved from purely inductive open coding to theory-informed pattern identification (Gioia *et al*., 2013; Pratt *et al*., 2020). Since we aimed at capturing the specific experience of the interviewees, we applied a reflexive thematic analysis to ensure a systematic coding and preserve researchers' reflexivity and sensitivity to the cultural context (Braun and Clarke, 2006, 2022; Clarke and Braun, 2017). The interview data were iteratively analyzed in an inductive-deductive manner to identify patterns and themes, with the aim of verifying and integrating the current evidence on team diversity with the collected data on experience.

Phase 1 - familiarization with the data – was carried out independently by two researchers who generated initial coding directly from the data. The analysis was carried out in Italian to ensure an adherent interpretation of the original transcripts and to preserve cultural and linguistic nuances that might be lost in translation during the analytical phase.

In Phase 2 - generating initial codes - the two researchers worked on a subset of interviews to develop an inductive preliminary codebook of recurring concepts. The segments of text were labelled if they were meaningful or relevant to the research aims, such as “alignment”, “diversity as a need”, “leader's role”, and “diverse goals in teams”. At the end of this step, the codebook was discussed to refine definitions and achieve a shared coding scheme to be applied to the rest of the material. Using the agreed-upon codebook, all transcripts were coded systematically, and new codes were added as they emerged from the interviews.

Phase 3 - constructing initial themes – and Phase 4 - reviewing and refining themes - engaged both researchers in the systematic coding and preliminary theme development from the entire collected material. The emerging codes were grouped into themes and sub-themes according to the research question. For example, codes related to the *“leader's role,” “functional leadership,” or “someone who wraps up” were grouped into the theme “The Supervisor's role” to represent* patterns of shared meaning among the respondents.

Finally, candidate themes were explicitly examined for alignment with or divergence from SCT predictions, thereby ensuring a dialogue between the inductive and deductive interpretations of the data. This sequence was chosen to ensure that our findings represented genuine patterns in the data rather than solely theoretical impositions. An example of coding is provided in Table 3.

**Table 3:** Example of coding

Piece of transcript	Initial code	Sub-theme	Theme
“You don't even choose diversity, but diversity chooses you. When you work in healthcare, it's normal to find yourself at a table discussing with people who have very different kinds of expertise.” (Interviewee 9)	Diversity as a need	Structural requirement for diverse expertise	Task-related diversity
“Create a good team within the district management, that is, have the ability to involve the various managers or various people … because you must have everyone's trust, and you must trust everyone, that is, you must know that you can delegate and you must have the ability to delegate, because if you want to make a team you must delegate.” (Interviewee 6)	Leader role	Building trust and delegation in team management	The supervisor's role

**Source(s):** Authors’ own work

In this paper, key quotations used to present and interpret our results were translated from Italian into English. Researchers were meticulous in preserving the meaning and tone of participants' words in translation. Quotations to support the description of results are presented with reference to the interviewee code.

## Results

The following paragraphs present the evidence emerging from the interviews with reference to the research question. The results are organized into six fundamental identified themes: (1) Task related diversity functionalities and dysfunctionalities, (2) Biodemographic diversity functionalities and dysfunctionalities, (3) Leaders vs Free riders, (4) External environment dynamics, (5) The supervisor's role, (6) The temporal relationship of team diversity, all conceptualized on the basis of SCT.

(1) Task-related diversity functionalities and dysfunctionalities

Overall, from the experiences of top and middle managers emerged a fundamental distinction between functional and dysfunctional diversity in teams. In their contexts, task-related diversity is already perceived not simply as a desirable feature but as a structural necessity.

You don’t even choose diversity, but diversity chooses you. When you work in healthcare, it's normal to find yourself at a table discussing with people who have very different kinds of expertise. (Interviewee 9).

We work towards complex objectives that require diverse skills. (Interviewee 6).

The increasing complexity of clinical, organizational, and societal challenges requires responses that no single profession can provide in isolation, necessitating collaboration across disciplines and sectors. Diversity is not merely chosen; it is inherently embedded in the work. This necessity, in line with the SCT, creates a context in which cross-professional observational learning becomes essential, as team members learn from one another's expertise to address complex healthcare challenges effectively. This is particularly evident in clinical decision-making processes, where professionals from diverse backgrounds, including physicians, nurses, technicians, therapists, social workers, and administrators, come together to deliberate and act jointly.

Interviewees provided several practical examples of how recognizing task-related diversity as a functional lever can help tackle complex challenges.

From the development of oncological screening programs requiring rigorous integration of clinical, organizational, and population-level strategies to the management of minors with psychiatric issues, where collaboration across healthcare, social services, and education is essential, the importance of diverse teamwork is evident. This principle also extends to structural and technological innovations, such as hospital design and the implementation of robotic surgery, where multidisciplinary teams ensure functional, safe, and efficient outcomes. (Interviewee 1).

These examples illustrate how positive expectations about diversity drive functional behaviors, as team members recognize that their collective efficacy depends on integrating diverse professional perspectives. Managers underlined how the COVID-19 pandemic offered a striking example of this necessity:

The level of care intensity has mostly increased due to COVID-19 … It has been possible to handle it thanks to integration and multidisciplinarity. (Interviewee 10).

These circumstances made visible what often remains implicit: that effective healthcare depends on the capacity to coordinate diverse tasks and perspectives coherently and dynamically. Indeed, the healthcare system can be understood as a system of communicating vessels based on a deep interdependence among roles.

Doctors cannot work without nurses. Nurses cannot work without doctors. The healthcare system can therefore be seen as a system of communicating vessels. (Interviewee 10).

The environmental pressure generated from COVID-19 created conditions in which social modeling of collaborative behavior became essential for survival, elucidating how external contexts can activate positive team mechanisms.

However, when social cognitive processes break down, the triadic interaction between personal factors, behavioral patterns, and environmental influence fails, and task-related diversity becomes dysfunctional. If all the pieces do not communicate in a synergistic and coherent way or do not share a common goal, what emerges is a disorganized or conflicting group of professionals. In such conditions, diversity loses its strategic value and may even become counterproductive.

Diversity becomes dysfunctional when there is no coherent and synergistic response to the complex problem at hand, and when the main objective is lost from view. In such cases, the value of diversity is diminished. (Interviewee 7).

(2) Biodemographic diversity functionalities and dysfunctionalities

Biodemographic diversity, encompassing factors such as age and gender, was not reported as a necessary condition for the basic functioning of healthcare teams. Surely, it can significantly impact organizational efficiency and team dynamics. In particular, the importance of age diversity in healthcare teams emerged as a recurring theme in the data. In fact, as the respondents recognized, young members can offer potential advantages, such as varied perspectives, complementary skills, and a richer pool of up-to-date ideas.

Younger professionals tend to be more inclined to innovate, bringing fresh approaches to longstanding problems. (Interviewee 8).

This, in line with SCT, highlights how positive outcome expectations about generational diversity can enhance team learning through observational learning processes in which different age groups model distinct problem-solving approaches.

Given that “*the solutions proposed to problems are always the same, so finding an innovative approach is difficult”* (Interviewee 9) involving younger people, especially in addressing complex challenges, was recognized as a valuable resource for team functionality. It is therefore *“important to work on the age diversity because we need to try to find different solutions*” (Interviewee 14).

Regarding gender diversity, most respondents emphasized that it is less relevant than task-related diversity, even though it can broaden perspectives and approaches to teamwork.

Gender diversity generates value to the team dynamics. (Interviewee 3).

The greater the gender diversity, the better, however, it is not a fundamental aspect. Since we are talking about healthcare professionals, I tend to look at this issue from the perspective of competencies. (Interviewee 2).

Despite these benefits, managers elucidated how biodemographic diversity also carries the risk of dysfunction if differences are not managed effectively. The mere presence of diverse demographic characteristics does not automatically translate into innovation or enhanced performance.

One of the main difficulties is making different generations coexist. (Interviewee 10).

Giving space to people with new ideas can also be a risk if there is no unity of purpose. (Interviewee 9).

From an SCT perspective, these tensions arise when team members develop negative self-efficacy beliefs about working across demographic differences and when environmental factors fail to support positive social modelling. In such situations, rather than serving as a source of strength, diversity becomes a barrier to effective teamwork. The managers underline this aspect in their direct experience.

When biodemographic diversity is forced and people are not able to find a point of contact, it leads to dysfunctional diversity. (Interviewee 3).

Age counts, because it immediately turns into prejudice of experience (Interviewee 1).

The dysfunction manifests here when negative outcome expectations about demographic differences become self-fulfilling prophecies, undermining the social cognitive processes necessary for collaborative learning.

(3) Leaders vs free riders inside the group

From the interviews, it emerged how diversification within teams, whether in expertise, bio-demographic characteristics, or perspectives, can generate different outcome expectations among the team members:

They didn't believe in the project, even though they were part of the team. This is the negative side the downside of our team at that moment … If they didn’t feel suited to the task or if it wasn’t the right fit for them, they needed to step back and make room for change. (Interviewee 2).

Hence, different expectations for team tasks and outcomes can undermine collective efficacy, leading to dysfunctional diversity. Failure to establish a cohesive team with a clear role dynamic can result in a *“group of people”* scenario, where individual members perform isolated tasks without real collaboration or obstacle the teamwork. This phenomenon, according to the experience of managers, may lead to pseudo-teams, formed solely to meet temporary objectives, such as securing funding, but lacking genuine cooperation and coordination over time:

There can be a person who does not believe in teamwork, who is not able to work in a team, who has personal problems with other team members. (Interviewee 12).

The challenge often lies in incorporating individual expectations with a shared sense of belonging (Interviewee 6).

In this scenario, free-riding and avoidance behaviors can emerge when individuals lack self-efficacy in contributing meaningfully to diverse teams.

In this regard, managers illustrated how forced inclusion in teams can suppress individual potential and create dysfunctional diversity dynamics.

I learned that forcing a person to be in a context or in a project where their contribution cannot be useful from the very beginning can have deep and complicated implications … if the group, in addition to being diverse in terms of expertise, is not unified in its purpose. … (Interviewee 2).

This highlights the necessity of diverse teams sharing common goals and members understanding “who is doing what” to avoid fragmented effort.

Moreover, resistance to change can foster free-riding behavior, wherein some members avoid contributing fully while criticizing others.

The free riding is due to the resistance to change, which often leads people to criticize others. (Interviewee 3).

we have some people that, you know, they feel uncomfortable to work in equipe. Their difficulties are so profound that if you ask them to accomplish a task, they come back saying I would like to do it in my way, but that way it is not possible, and you do not get back a solution (Interviewee 6).

(4) The Supervisor's role

The role of the supervisor emerged as one of the most cited themes, with great emphasis on the need for *“someone who wraps up”*. The supervisor within a team can deeply influence the group's cohesion, effectiveness, and ability to address challenges.

The role of the supervisor is fundamental when they have to create synthesis, but it is not a role you can simply appoint yourself to; others must recognize it, and not everyone has the ability to do so. (Interviewee 6).

The analysis shows that managers intend coordination not as a mere title or formal position; it requires recognition and acceptance by team members, as well as specific skills for the supervisor that not everyone inherently possesses. In fact, they underlined how effective supervisors act as both leaders and facilitators, fostering an environment where concerns and challenges can be openly addressed and strategies are shared.

The supervisor of the group has greater responsibilities, including keeping the team cohesive and understanding when there are issues. It’s important for the supervisor to keep the door open for any necessary listening to understand the problems. (Interviewee 1).

Accordingly, without clear leadership and direction, diversity can become a source of fragmentation rather than a source of strength. The supervisor ensures that the team's outcome expectations are aligned and that diversity contributes constructively to achieving shared goals.

The supervisors are fundamental because they provide direction; without a clear direction, even a well-diversified team cannot effectively respond to problems. (Interviewee 3).

Further to this topic, managers reported that leadership effectiveness depends on team members' beliefs about the supervisor's ability to enhance collective efficacy.

Specifically, in their experience with the healthcare sector, the supervisors serve as a linchpin that holds the team together. Their effectiveness is not solely a matter of personal authority but depends significantly on their relational competence and their ability to adapt their leadership style to the team's structure and needs.

In my experience, I adopted different types of leadership depending on the team context, ranging from purely supportive roles to necessarily authoritative ones. (Interviewee 12).

This adaptive approach underscores the need for supportive factors to align with team characteristics to optimize social-cognitive processes and collective efficacy. Thus, managing team dynamics in healthcare requires more than assembling diverse individuals; it demands a deliberate alignment of goals, roles, and interpersonal relationships. Hence, only by being managed can diversity fulfil its promise as a driver of innovation and effective care delivery, rather than becoming a source of conflict and dysfunction that exacerbates dysfunctional behaviors.

(5) External environment dynamics

The collected evidence shows that analyzing team diversity without considering the external environment in which these groups operate can yield a limited and sometimes misleading perspective on their effectiveness.

Not considering the external environment is limiting because teams that might be optimal in their composition can then clash with everyday realities such as slow bureaucracy. (Interviewee 1).

The tension between the group's internal needs and external rigidities can thus generate inefficiencies or conflicts. On the other hand, the external environment can also represent an opportunity to support and enhance internal diversity. In this respect, the respondents highlighted how teams that might initially appear suboptimal in their composition may benefit from a favorable context that supports their work.

On one hand, the challenge lies in the external environment, such as insufficient funding that hampers project completion. On the other hand, diversity remains crucial because a well-diversified team can effectively manage different aspects of the project, unlike a diverse but dysfunctional team. (Interviewee 12).

In the same vein, another factor that can complicate team management is the involvement of the population or citizens in team activities, often required by relevant regulations. On the one hand, managers recognized that including diverse perspectives and expertise can enrich decision-making processes and improve outcomes. On the other hand, they reported how involving external actors means managing expectations, communication, and negotiation, which can slow down and complicate the team's work.

The involvement of the population as required by relevant regulations clearly means that often in work groups we also involve citizens, and this complicates things. It brings advantages but also complications. (Interviewee 6).

This external requirement introduces additional social-cognitive complexity, as diverse external stakeholders can affect the individual's self-efficacy and outcome expectations, thus impacting on team functionality.

(6) The temporal dynamics of team diversity

Teams are inherently dynamic entities that evolve over time, adapting to changing objectives, fluctuating membership, and shifting contextual demands. Therefore, diversity benefits do not emerge automatically or immediately; instead, they are deeply contingent on the temporal dynamics within which teams operate. Managers viewed an initial alignment phase as crucial for establishing and clearly defining specific roles, responsibilities, and tasks among group members. Without this phase, diversity risks becoming a source of misunderstanding, conflict, and inefficiency rather than a driver of efficiency.

If you want to achieve results … you must allow the group to adapt. (Interviewee 2).

The group evolves over time. (Interviewee 6).

This temporal evolution reflects how collective efficacy and positive outcome expectations develop through repeated successful experiences and observational learning.

In this process, trust emerges as one of the primary characteristics that guarantee the functionality of diverse teams.

As trust develops reciprocally among team members, it becomes the essential social glue that enables open communication, mutual respect, and effective collaboration … In diverse teams, where differences in background, expertise, and status can lead to misunderstandings and misalignments, fostering transparency, continuous dialogue, and shared goals is critical to ensuring functional teamwork. (Interviewee 1).

Yet, the process of integrating diversity is rarely straightforward. The introduction of new professional figures often creates initial disruption and adjustment challenges. In healthcare, for instance, managers have illustrated that accepting different professions providing complementary support can be fraught with difficulties, including role ambiguity and resistance from established members.

However, at the same time, one of the main difficulties is dealing with the change that the introduction of new professional figures can create. So there is also this difficulty in healthcare of accepting different professions that can provide support. (Interviewee 6).

In the subsequent phases, once the team has established a foundation of trust and transparency, diversity can be leveraged more effectively, enabling the team to benefit from a broader range of perspectives and expertise.

In the second phase, communication, transparency, mutual trust, and discussion are needed to ensure that everyone shares the same goals and competencies, especially in diversified teams (Interviewee 1).

Consequently, it is important to consider the temporal phase at which the team evaluates the effects of diversity. Newly formed teams or those undergoing significant changes may require more time, effort, and structured support to harness the advantages of diversity. Conversely, managers confirmed that teams with an established history and well-integrated members tend to experience faster and more positive returns from diversity, as they have already navigated early-stage challenges. In this sense, the triadic interaction between personal factors, behavioral patterns, and environmental influence may require time to become synergistic.

### Suggestions for the management of diversity

Effective diversity management in organizations hinges on several critical levers that shape team diversity dynamics. This paragraph summarizes specific suggestions and best practices elicited from the interviews.

Among the available levers for improving diversity management, a change in membership or roles may be necessary to restore group functionality and advance collective objectives. This perspective underscores the dynamic nature of teams, where restructuring is not a sign of failure but a strategic lever to maintain effectiveness.

A group needs sometimes to be rebuilt (Interviewee 9).

Sometimes teams need to be restructured based on changing goals. Turnover should be natural, not forced. (Interviewee 2).

Managers' experience reflects an understanding that team evolution should align with shifting organizational priorities and market demands, and that, when possible, thoughtful management of turnover serves as a mechanism to refresh outcome expectations and collective efficacy.

Turnover brings fresh energy even in good teams. New people introduce ideas, even informally. But change is hard in rigid hierarchies. The key levers are relationships, open dialogue, courage to disagree, and competence. (Interviewee 14).

Turnover is positive when it leads to elements aiming to generate new energy in the team. (Interviewee 3).

Beyond turnover, internal communication mechanisms were reported as vital levers. These forums create space for dialogue and collaborative problem-solving, enhancing inclusion and shared ownership of outcomes.

Internal meetings are crucial for team members to discuss problems and find joint solutions. (Interviewee 7).

Involving all team members in decisions, regardless of rank, is necessary. (Interviewee 4).

Setting clear objectives and providing training were also reported as fundamental to managing diversity effectively, highlighting the importance of aligning diversity efforts with organizational goals and investing in continuous development to equip teams with the skills needed to operate inclusively, and fostering an organizationally diverse-oriented culture.

Past experience has shown that well-structured goal systems and engaging learning experiences help teams grow. (Interviewee 14).

… to foster an organizational culture that encourages behaviors aimed at valuing diversity. (Interviewee 8).

Furthermore, managers identified that operational practices such as job rotation and flexible contracts can actively support diversity. This approach moves beyond rigid hierarchical structures, promoting meritocracy and opening opportunities for diverse talents to emerge and contribute.

Encouraging exchanges, job rotation, and greater flexibility in contracts so that selection is based not on hierarchical hiring but on identifying the most needed skills from the list of eligible candidates could foster diversity and performance. (Interviewee 15).

## Discussion and conclusion

The presented study contributes to the literature by examining the experiences of middle and top managers regarding the functionality or dysfunctionality of diversity and by identifying the key drivers of diversity in a complex sector, such as healthcare. Social Cognitive Theory was referred to as integrating inductive interpretation of data with examination of the practical, contextual reality of healthcare settings, and analyzing the functional and dysfunctional aspects of diversity in teams. Indeed, SCT allows us to elucidate how diversity, in light of contextual factors, can manifest functionally, aligning with positivist theories such as resource dependence theory and information/decision-making theory, or negatively, as predicted by social identity theory's subgroup conflict dynamics. Building upon the SCT, the study offers a practical, evidence-based contextual framework that can be replicated in other sectors and used by human resources managers and by team leaders in decision-making processes related to team diversification. The presented results support the importance of purposively managing diversity in teams to ensure their functionality and prevent them from becoming dysfunctional groups (Peduzzi, 2001). In the direct experience of managers, the multiprofessional component, related to task-related diversity, is fundamental to achieving the objectives and is therefore consistent with Horwitz and Horwitz (2007), constituting an essential element of the team that would otherwise be unable to tackle the wicked problems the healthcare sector is called upon to address. The biodemographic component, on the other hand, acts as a non-fundamental but crucial factor. In particular, age diversity emerges as both a source of potential conflict to be managed and the main element capable of fostering innovation. In general, all biodemographic components are regarded as drivers for managers to integrate new solutions and fresh perspectives when the group is trained in inclusion.

Through the lens of SCT, these dynamics can be understood as manifestations of reciprocal determinism, where personal factors, behavioral patterns and environmental influences continuously interact to shape team outcomes (Bandura, 2001). The examined experience of diversity within healthcare teams reflects a complex interplay among these three determinants, in which individual cognitive and social characteristics influence and are influenced by team behaviors and the broader organizational environment.

In this sense, all the interviews provide evidence to support the value of differentiated human and social capital within the team, broadening the team's resources, viewpoints, and actions (Bantel and Jackson, 1989; Hillman *et al*., 2009; Salancik and Pfeffer, 1978; van Knippenberg *et al*., 2004). However, it should be noted that diversifying a team *per se* is not sufficient to guarantee the functionality of diversification, since diversity can also be dysfunctional. In fact, the presence of diverse competences, habits, languages, and viewpoints, when combined, positively impacts team outcomes only if team members feel part of the team and agree on the overall objectives to be pursued together. This aligns with SCT's emphasis on outcome expectations and collective efficacy, where shared beliefs about the group's capabilities to perform effectively are crucial for successful collaboration (Bandura, 2000). Specifically, one of the main aspects that we aim at conceptualizing is the unintended consequences of diversity (Von Bergen *et al*., 2002). Hence, an excessive level of diversity within groups, regardless of whether it is biodemographic or task-related, can have a counterproductive effect if not properly managed (Mitchell *et al*., 2022).

**Figure 1:**
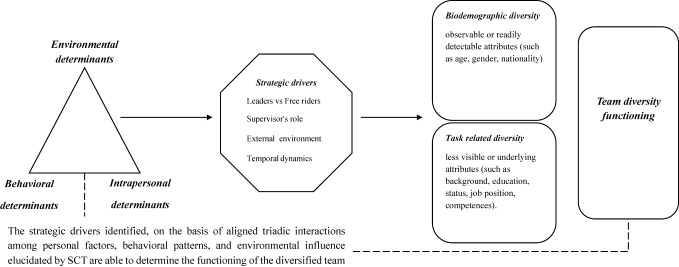
Strategic drivers for team diversity functioning

In this context, several contextual factors, as illustrated in Figure 1, can determine the functioning of the diversified teams on the basis of the triadic social cognitive interactions.

In line with other studies, the interviews reveal the importance of a clear unity of purpose and consistent collaboration among team members (Dreachslin *et al*., 2000). Alternatively, the tendency of some members to benefit from others' efforts without adequately contributing, a behavior often fueled by resistance to change, leads to what is known as free riding (Glover and Kim, 2021; Hütter and Diehl, 2011), which has critical effects on the collaborative climate and can significantly reduce team effectiveness, particularly in complex settings such as healthcare. Hence, free-riding behavior can be understood as a result of low self-efficacy beliefs regarding one's ability to contribute meaningfully to team objectives, combined with negative outcome expectations about the consequences of active participation (Bandura, 1997). In this context, diversity may play a dysfunctional role, accelerating this dynamic. Difficulties in communication and collaboration among team members may lead to the formation of the so-called “pseudo-teams” groups of individuals working in isolation without genuine cooperation (West and Lyubovnikova, 2012, 2013). A striking example of such tensions is the inability to integrate different professional perspectives within a healthcare team (Salas-Vallina *et al*., 2017). This highlights the need not only for diversity but also for the sharing of common goals and a clear definition of roles and responsibilities in order to avoid fragmentation and counterproductive free-riding behaviors that threaten organizational well-being. Within this scenario, the supervisor's crucial role becomes particularly evident. Supervisors serve as critical models for collaborative behavior through observational learning processes, where team members observe and potentially imitate supervisory behaviors related to conflict resolution, inclusive decision-making, and diversity management (Bandura, 1977, 2001). The supervisor can significantly influence group cohesion, team effectiveness, and the ability to face complex challenges. It serves as a convergent point for diverse perspectives and synthesizes individuals, guiding the team toward a shared direction (Griffin *et al*., 2001; Mathieu *et al*., 2016). Additionally, effective supervisors enhance team members' collective self-efficacy by providing supportive feedback, creating opportunities for successful experiences with diversity, and modelling confident leadership behaviors that underline how diverse perspectives can be integrated productively (Bandura, 2000; Yukl, 2012).

Specifically, in the bottom-up teams, typical of the healthcare sector the supervisor's role tends to shift towards that of a mediator and facilitator, supporting the group in internal negotiation and in the autonomous management of decision-making processes which is also linked to professional bureaucracy of the healthcare sector, where doctors and other health professionals are highly educated and competent, and thus a figure is needed to mediate between the various perspectives and bring them together into a shared decision (Gibson and Vermeulen, 2003; Jehn and Mannix, 2001; Kozlowski and Ilgen, 2006).

Hence, another strategic aspect that emerged from the interviews is the importance of the external environment. In this sense, the SCT reciprocal determinism framework emphasizes that environmental factors significantly influence both individual cognitions and behaviors within teams (Bandura, 2001). Analyzing team diversity without considering the external context in which such teams operate results in an inevitably partial and potentially misleading perspective. In fact, while a positive external environment can foster diversity and inclusion (Ashikali and Groeneveld, 2015), critical environmental factors can hinder decision-making and slow the implementation of activities. This is especially evident in public, healthcare, or heavily regulated organizations, where administrative and regulatory constraints may clash with the flexibility and decision-making speed required by heterogeneous teams, negatively impacting team collective efficacy and the general climate of the team (Hicks-Clarke and Iles, 2000; Jaiswal and Dyaram, 2019; Lee *et al*., 2021).

Moreover, another key driver that emerges is time. The benefits derived from diversity within teams are neither immediate nor guaranteed but develop over time through complex evolutionary dynamics (Harrison *et al*., 2002; Kozlowski and Ilgen, 2006).

Tuckman (1965) highlights how teams go through different stages: forming, storming, norming, and performing and that only after an initial period of misalignment and critical confrontation (storming) can effective collaboration be achieved (norming and performing). These stages represent progressive development of collective efficacy and refined outcome expectations, as team members learn through experience how to coordinate their diverse contributions effectively (Bandura, 2000). This temporal evolution underscores the importance of supporting a gradual increase in the group efficacy of heterogeneous team members. Contrary to a static view, the analyses reveal the dynamic nature of teams, in which restructuring the group through changes in composition or roles can be a strategy for managers to foster adaptation and performance renewal, rather than an indicator of failure (Arrow *et al*., 2000; Mathieu *et al*., 2014). Indeed, if one or more members change, the group needs an adjusting phase of internal realignment that negatively impacts its effectiveness (Tuckman, 1965).

Finally, enriching the policy and practice implications, managers also recommended several best practices for managing diversity. In this sense, the use of turnover, seen as a strategic rotation of team members, can be considered as an important lever for renewing skills, stimulating critical thinking, and preventing dysfunctionalities. However, in contexts characterized by rigid hierarchical structures or organizational cultures oriented towards stability, such as the healthcare sector, change is often perceived as a threat rather than an opportunity (Hom *et al*., 2019; Poon *et al*., 2022). Another key element is the quality of internal communication mechanisms. The presence of structured spaces for discussion, such as team meetings, workshops, or moments of reflection-in-action, promotes dialogue, active involvement of all members, and the co-construction of solutions. These communication structures facilitate observational learning and provide opportunities for healthcare team members to observe successful diversity management behaviors, gradually building their confidence and skills in working across differences. Clarity of objectives and the availability of targeted training paths also represent essential levers. At the organizational level, building a culture that values diversity is a transversal lever that can profoundly influence behavioral norms and shared expectations. In this line, Ely and Thomas (2001) show how organizations adopting an integration-and-learning perspective are able to embed diversity into daily processes, promoting innovation and continuous improvement. This culture translates into concrete practices such as job rotation, flexible contracts, and merit-based recognition.

This research has some limitations. First, while the study offers valuable insights within the healthcare sector, the results may not be directly applicable to all types of teams or industries, as team dynamics can vary significantly across different contexts. The focus on the Italian healthcare system further limits the external validity of the findings: healthcare organizations in Italy operate within specific cultural, institutional, and regulatory frameworks, which may not be representative of other countries or cultural settings. Moreover, potential biases in data coding and translation cannot be entirely excluded. Since the analysis involved interviews conducted in Italian and later translated into English for coding and interpretation, subtle linguistic nuances or cultural meanings might have been lost or slightly altered during translation. Additionally, the study does not consider how cultural differences may influence the effectiveness of diverse teams. Cultural factors can significantly shape team interactions, especially in multinational or culturally diverse settings. Consequently, the results may not be directly transferable to other sectors or national contexts where institutional dynamics, professional roles, and cultural values differ. Team diversity and interaction patterns can be influenced by sectoral characteristics, such as hierarchy, decision-making processes, and communication norms, as well as by broader cultural dimensions. Moreover, because the data were collected from a purposive sample, the findings may reflect the personal biases and perceptions of the interviewees, potentially introducing subjectivity and not always reflecting the broader group of healthcare managers.

Future research could add further evidence to confirm or more precisely specify the analysis presented, involving employees from other contexts or countries.

While SCT provides valuable insights into the cognitive and behavioral mechanisms, future research could also benefit from more systematic measurement of self-efficacy beliefs, outcome expectations, and observational learning processes within diverse teams.

The study should be replicated in a differentiated sample, to be reached across different settings and industries. It would also be valuable to explore the impact of cultural differences on team functionality, particularly in multicultural environments, to understand how diversity is managed in global or cross-cultural teams. Future research could investigate how the identified drivers of functional and dysfunctional diversity apply or vary across public and private healthcare systems, analyzing the factors that contribute to the creation of pseudo-diversified teams. Additionally, incorporating quantitative data could provide a more comprehensive view, adding robustness to the qualitative findings thanks to triangulation. A longitudinal approach could also be considered to examine the long-term effects of team diversity on organizational success and team cohesion to better analyze the temporal dimension of team diversity. Such longitudinal studies would be particularly valuable for testing SCT predictions about how cognitive processes and behavioral patterns evolve over time in diverse teams. Therefore, exploring the role of leadership in managing team diversity and mitigating potential conflicts would further enrich our understanding of how diverse teams can be successfully managed. Finally, this research opens a call for articles conceptualizing the strategic drivers of diversity and the strategic solutions to its counterproductive consequences, and for studies analyzing the effective functioning of teams.
